# A Case of Sarcoidosis Diagnosed Based on Progressive Renal Dysfunction and Lymphadenopathy

**DOI:** 10.7759/cureus.83936

**Published:** 2025-05-11

**Authors:** Tomohide Sato

**Affiliations:** 1 Division of Cardiology, Saiseikai Kumamoto Hospital Cardiovascular Center, Kumamoto, JPN

**Keywords:** axillary lymphadenopathy, heart failure with preserved ejection fraction, non-caseating epithelioid cell granuloma, renal failure progression, renal sarcoidosis

## Abstract

Several series have suggested that kidney involvement (as defined by either histological changes in the kidney or a decline in kidney function in the absence of a biopsy) occurs in approximately 10 to 50 percent of patients with sarcoidosis. A large study examining more than 1200 hospitalized patients with sarcoidosis reported that kidney manifestations were present in approximately 6% of cases. The case is that of an 89-year-old woman. She experienced paroxysmal nocturnal dyspnea starting four days before her visit. Three days before coming to our hospital, she consulted a nearby clinic, leading to a suspicion of heart failure, and she was referred for further evaluation. Physical examination revealed left axillary lymphadenopathy, coarse crackles in both lung fields, and slow pitting edema over both tibial tuberosities. Blood tests showed Cr: 2.10 mg/dL and B-type natriuretic peptide (BNP): 1144.4 pg/mL. The sIL-2R obtained for the purpose of differentiating malignant lymphoma was 3290 U/mL and elevated. A plain chest CT scan showed cardiomegaly, pulmonary edema, pleural effusion, and left axillary lymphadenopathy. Based on subjective symptoms, physical findings, and laboratory test results, the patient was diagnosed with an acute exacerbation of chronic heart failure. Treatment with atrial natriuretic peptide and loop diuretics was started on the same day for heart failure. Her dyspnea gradually improved, and her weight also decreased. Radiographic findings also showed an improvement in pulmonary congestion. However, by the 14th day of hospitalization, her creatinine level had worsened to 5.85 mg/dL, indicating renal dysfunction. Due to persistent lymphadenopathy and rapidly progressing renal dysfunction, renal sarcoidosis was suspected, and the patient was transferred to another hospital for further investigation. A lymph node biopsy from the left inguinal region revealed non-caseating epithelioid cell granulomas. The serum lysozyme level, which has been shown to be elevated in cardiac and renal sarcoidosis, was 30.6 μg/mL. The diagnosis was sarcoidosis, with heart failure and renal failure attributed to cardiac sarcoidosis and granulomatous interstitial nephritis. Maintenance dialysis was decided upon for renal dysfunction. Sarcoidosis is a systemic disease characterized by the appearance of non-caseating epithelioid cell granulomas. Organs such as the heart, kidneys, eyes, lungs, and nervous system, which impact quality of life and prognosis, require careful monitoring over time. Lymphadenopathy, which does not align with the usual course of heart failure, and progressive renal dysfunction despite achieving diuretic effects, should raise suspicion for this condition.

## Introduction

Sarcoidosis is an idiopathic multisystem disorder affecting organs such as the heart, kidneys, lungs, and lymph nodes, characterized by the accumulation of activated T lymphocytes, mononuclear phagocytes, and noncaseating granulomas in the affected tissues [1,2]. Although this disease has traditionally been considered common in young to middle-aged adults, recent data suggest that it may also occur in individuals in their 60s and 70s, especially among women. Approximately 90% of patients have pulmonary involvement, with most of the morbidity and mortality associated with this disease resulting from pulmonary disease. However, clinically significant kidney involvement is occasionally observed. Kidney manifestations include interstitial nephritis and nephrocalcinosis or nephrolithiasis due to abnormal calcium metabolism. The typical kidney lesion is noncaseating granulomatous interstitial nephritis. When renal dysfunction progresses rapidly despite improvements in various parameters due to treatment, or when lymph nodes are palpable, this condition should be considered. Glomerular disease, obstructive uropathy, and end-stage kidney disease (ESKD) may also occur, although these are rare [3,4]. In elderly patients with concurrent cardiac and renal sarcoidosis, a definitive diagnosis requires a biopsy. However, in older individuals, the risks of complications and other factors make performing a biopsy more challenging, potentially leading to a delayed diagnosis. Here, we report a case involving an older patient who simultaneously developed cardiac and renal sarcoidosis.

## Case presentation

The patient was an 89-year-old female who presented to our hospital with a chief complaint of paroxysmal nocturnal dyspnea. Her medical history includes hypertension, gastroesophageal reflux disease (GERD), chronic constipation, and insomnia. There was no history of autoimmune diseases or granulomatous disorders. Her current regular medications are as follows: alacepril 100 mg/day, cilnidipine 20 mg/day, furosemide 20 mg/day, doxazosin mesilate 2 mg/day, rabeprazole sodium 10 mg/day, rebamipide 200 mg/day, Marzulene S (a gastrointestinal protectant) 4.5 g/day, senna 0.5 g/day, and lormetazepam 0.1 mg/day. There are no significant findings in her family medical history. Five days prior to her hospital visit, she developed a wet cough. Four days prior, she experienced the onset of paroxysmal nocturnal dyspnea. Three days prior, she consulted a nearby physician, where blood tests revealed a significantly elevated B-type natriuretic peptide (BNP) level of 1699 pg/mL, indicating possible cardiac failure, leading to her referral to our hospital. The patient did not present with persistent fever, night sweats, or weight loss. There was no indication of excessive salt intake or medication non-compliance, and she denied any recent infectious illnesses. There was no fever, night sweats, or weight loss. Upon arrival, the patient's vital signs were as follows: she was alert and oriented, with a height of 128 cm, weight of 44.8 kg, body temperature of 37.1°C, respiratory rate of 12 breaths per minute, blood pressure of 130/65 mmHg, heart rate of 83 beats per minute, and an oxygen saturation (SpO2) of 98% on room air. Physical examination revealed no pallor of the palpebral conjunctiva and no jaundice of the sclera. A lymph node enlargement of approximately 5 cm was palpated in the left axilla. A holosystolic murmur was audible at the apex of the heart. Coarse crackles were heard throughout both lung fields. Slow pitting edema was noted over both tibial tuberosities. The patient has normocytic anemia with an Hb level of 8.1 g/dL and a mean corpuscular volume (MCV) of 93.0 fL. Hypoalbuminemia is also observed, with an albumin level of 3.6 g/dL. Severe renal dysfunction is indicated by a serum creatinine level of 2.1 mg/dL. BNP (1144.4 pg/mL) and soluble interleukin-2 receptor (sIL-2R) (3290 U/mL) are markedly elevated.

Blood tests

The blood test results are shown in Table 1.

**Table 1:** Blood tests on admission BNP: B-type natriuretic peptide

Tests performed	Result	Reference range
White blood cells (U/mL)	3290	3100~8400
Neutrophils (%)	65.9	40-75
Lymphocytes (%)	24.0	20-50
Monocytes (%)	5.4	2-10
Eosinophils (%)	4.40	0-6
Basophils (%)	0.30	0-3
Hemoglobin (g/dL)	8.1	14.0-17.5
Platelet count (×10^4^/μL)	27.8	15.6-37.3
Total protein (g/dL)	6.6	6.5-8.5
Albumin (g/dL)	3.6	4.0-5.2
Sodium (mEq/L)	143	135-145
Potassium (mEq/L)	4.1	3.5-5.0
Chloride (mEq/L)	111	96-107
Calcium (mg/dL)	7.8	8.8-10.6
Blood urea nitrogen (mg/dL)	89	9-21
Creatinine (mg/dL)	2.10	0.5-0.8
Glucose (mg/dL)	135	65-109
Glutamic-oxaloacetic transaminase (IU/L)	14	5-37
Glutamic-pyruvic transaminase (IU/L)	9	6-43
Lactate dehydrogenase (IU/L)	247	124-222
Total bilirubin (mg/dL)	0.43	0.4-1.2
Alkaline phosphatase (IU/L)	263	38-113
Gamma-glutamyl transferase (IU/L)	25	0-75
Total cholesterol (mg/dL)	148	150-219
High-density lipoprotein cholesterol (mg/dL)	43	45-75
Low-density lipoprotein cholesterol (mg/dL)	87	70-139
Triglycerides (mg/dL)	79	30-149
C-reactive protein (mg/dL)	4.2	<0.30
BNP (pg/mL)	1144.4	≦18.4
Glycated hemoglobin (%)	6.00	4.6-6.2
Thyroid-stimulating hormone (μgIU/mL)	5.0196	0.5-5.00
Free triiodothyronine (pg/mL)	1.61	2.4-4.5
Free thyroxine (ng/dL)	1.05	1.0-1.7
Soluble interleukin-2 receptor (U/mL)	3290	122-496

Urine tests

The results of the urinalysis are shown in Table 2. Protein/microalbumin excretion and granular casts are observed.

**Table 2:** Urine tests on admission RBC: Red blood cells, WBC: White blood cells

Tests performed	Result	Reference range
pH	6.5	5.0-80
Glucose	2＋	(- ~ ±)
Protein	2＋	(- ~ ±)
Occult blood	1＋	(-)
Urobilinogen (EU/dL)	normal	0.1~1.0
Ketone bodies	negative	(-)
Urinary sediment analysis		
RBC (/HPF)	5-9	<5
WBC (/HPF)	20-29	<5
Squamous epithelium (/HPF)	5-9	<1
Granular casts (/HPF)	>100	0
Bacteria	1＋	(-)
Macroalbumin	3＋	(-)

Electrocardiogram

A 12-lead electrocardiogram revealed sinus rhythm, normal axis deviation, and a heart rate of 86/min. No obvious blocks were observed.

Plain chest computed tomography (CT)

**Figure 1:**
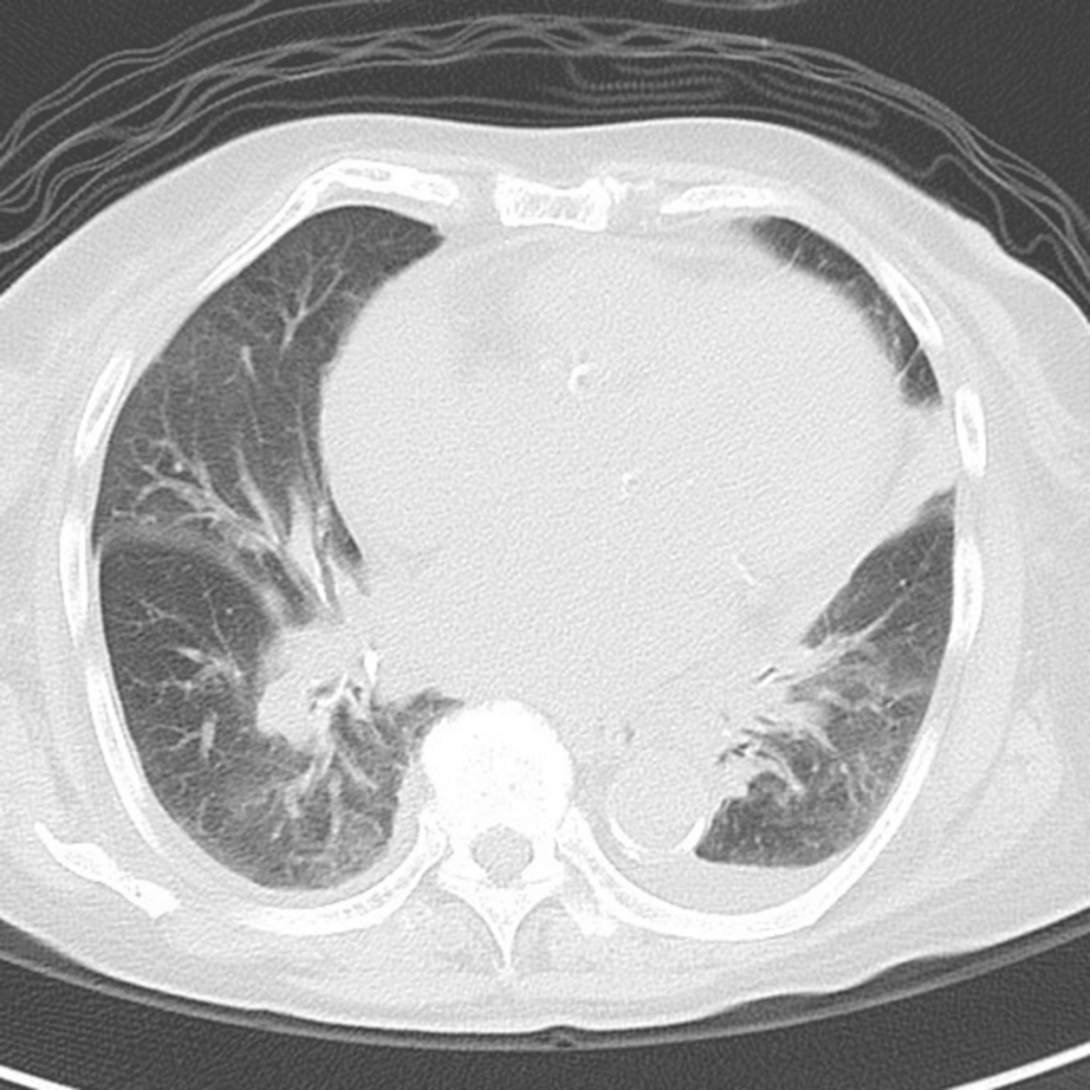
Plain chest computed tomography (CT) Plain chest CT revealed cardiac dilation, pulmonary edema, and mild pleural effusion.

**Figure 2:**
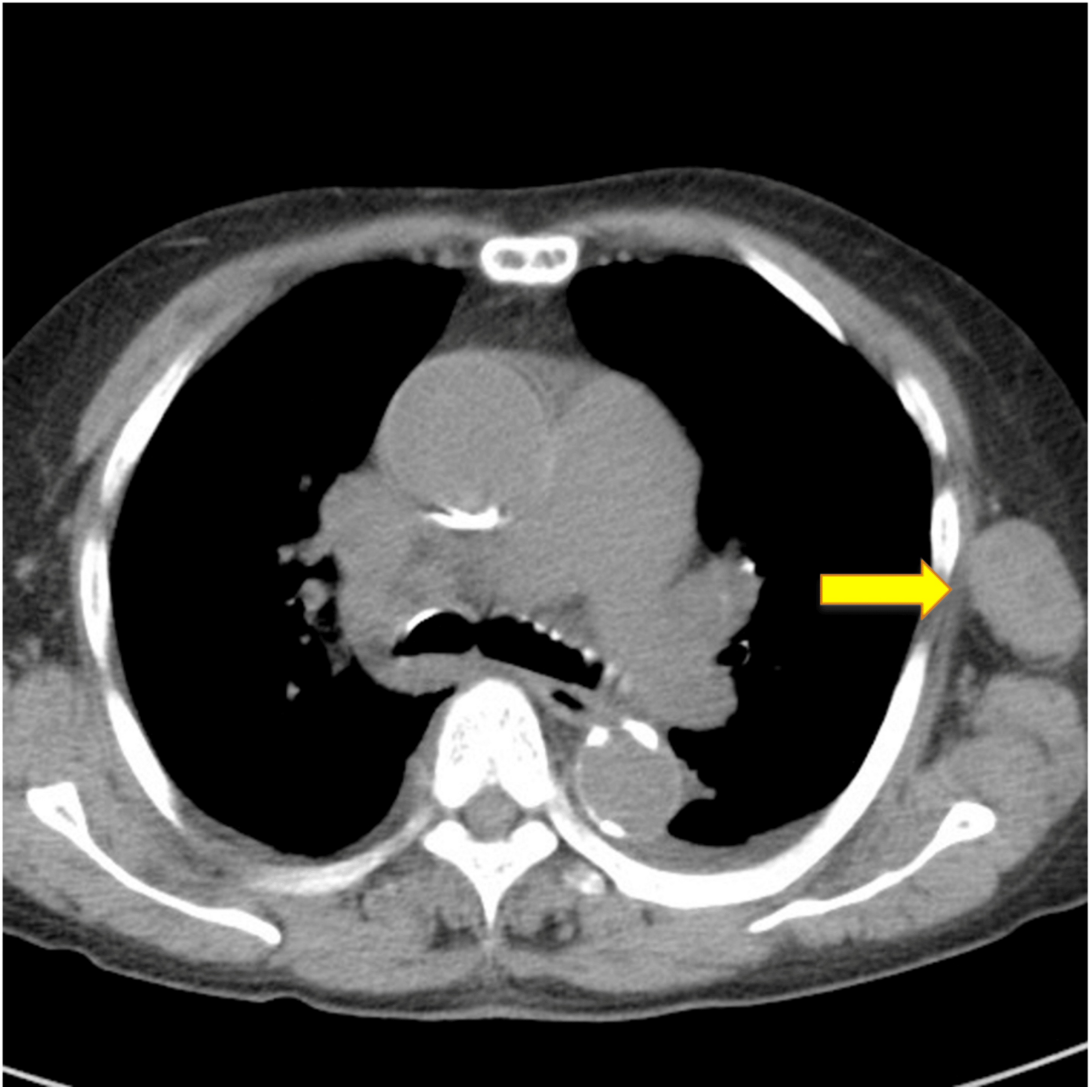
Plain chest computed tomography (CT) Swelling of the left axillary lymph nodes were observed.

Plain chest CT revealed cardiac dilation, pulmonary edema, mild pleural effusion, and swelling of the left axillary lymph nodes (Figures 1, 2).

Transthoracic echocardiography

The ejection fraction was 56.3%, the E/e' ratio was 22.1, and moderate mitral regurgitation was observed, suggesting diastolic dysfunction. The inferior vena cava diameter ranged from 6.6 to 15.5 mm. Pericardial effusion was 6.0 mm. The thinning of the base of the septum could not be identified.

Abdominal ultrasound

**Figure 3:**
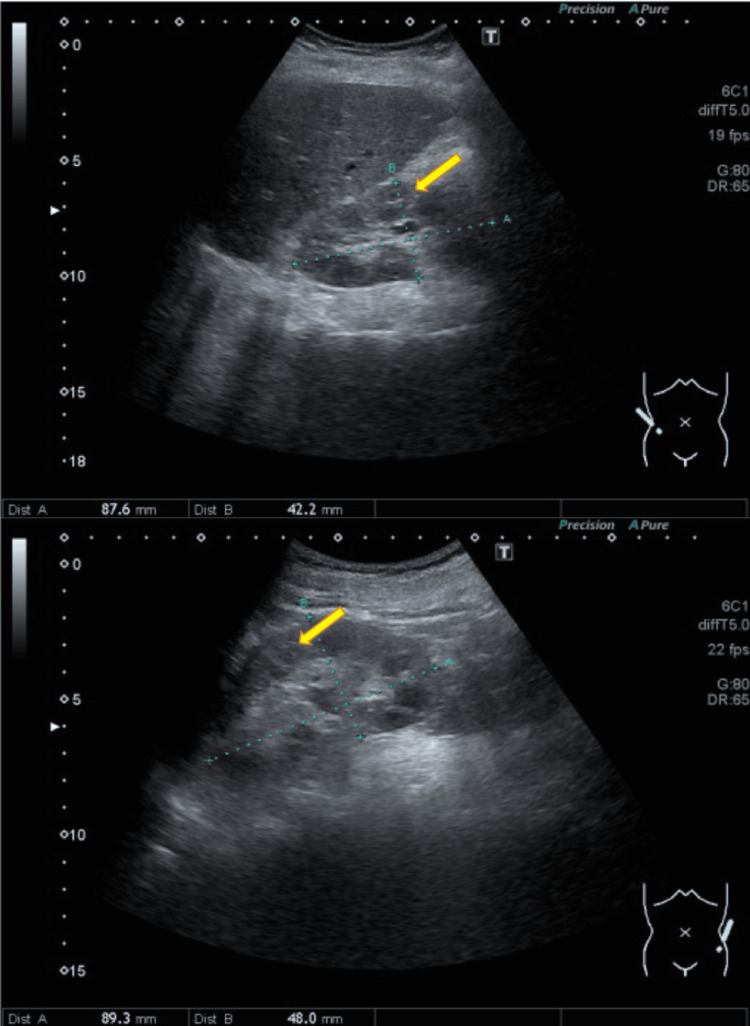
Abdominal ultrasound Here is the abdominal ultrasound examination: The upper panel shows the right kidney, and the lower panel shows the left kidney. There is a slight increase in echogenicity in both renal cortices (indicated by yellow arrows). No abnormalities in blood flow or morphology were detected.

Abdominal ultrasound revealed slightly increased echogenicity in both renal cortices. No abnormalities in blood flow or shape were detected (Figure 3).

**Figure 4:**
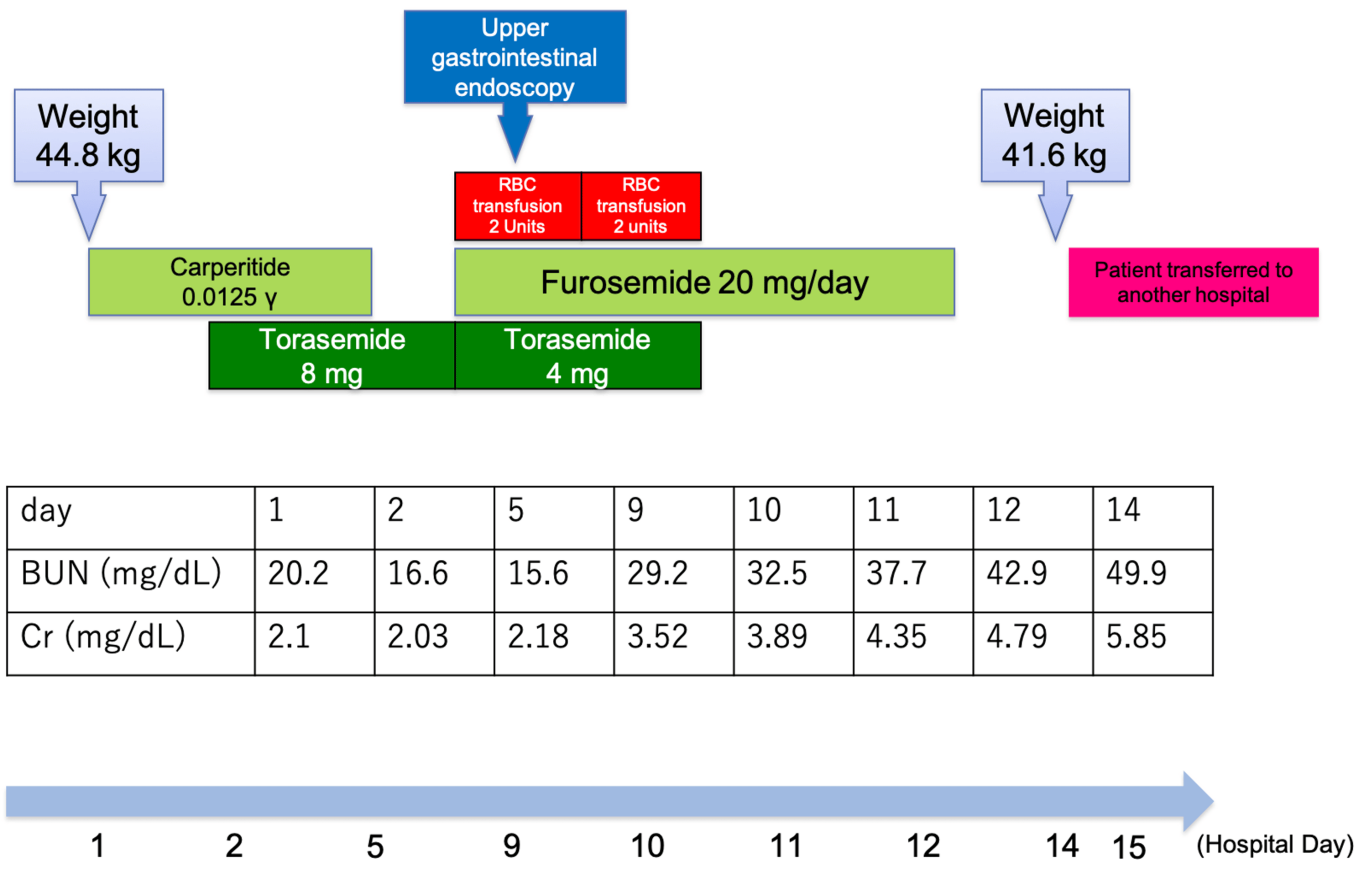
Course during hospitalization RBC: Red blood cell, BUN: Blood urea nitrogen, Cr: Creatinine.

The patient’s hospitalization course (Figure 4) is mentioned below. Based on the assessment of lung sounds, lower leg edema, elevated BNP levels in blood tests, and the presence of cardiac dilation and pulmonary edema on imaging, a diagnosis of cardiac failure was established. The echocardiogram indicated that there was no significant reduction in wall motion, and contractile function was preserved. Consequently, cardiac failure with preserved systolic function was diagnosed, and treatment with intravenous carperitide at a dosage of 0.0125 γ was initiated. Regarding the left axillary lymph node enlargement observed during physical examination and imaging, further evaluation was planned to occur after the symptoms of heart failure had subsided. On hospital day two, the patient began an oral regimen of torasemide at 8 mg/day. Subsequently, improvements were noted in nocturnal dyspnea, with urine output reaching 1700 mL/day, which was subsequently maintained. A chest X-ray performed on the same day demonstrated a reduction in signs of congestion, and the administration of carperitide was completed. However, on hospital day nine, blood tests revealed blood urea nitrogen at 29.2 mg/dL, Cr at 3.52 mg/dL, and Hb at 7.4 g/dL, indicating a progression of renal dysfunction and anemia. An emergency upper gastrointestinal endoscopy was conducted to investigate the anemia, but no significant bleeding was observed. The patient received two units of RBC transfusion and was administered 20 mg of furosemide, while the dosage of torasemide was reduced to 4 mg/day. By hospital day 10, Hb improved to 9.1 g/dL; however, renal function deteriorated further, with blood urea nitrogen (BUN) rising to 32.5 mg/dL and Cr to 3.89 mg/dL. Drug-induced renal dysfunction was suspected, prompting the discontinuation of all oral medications. The patient received another two units of RBC transfusion, along with 20 mg of furosemide. Despite the cessation of oral medications, renal dysfunction continued to progress gradually. Blood tests conducted two months prior to hospitalization showed BUN at 17.3 mg/dL and Cr at 0.66 mg/dL, suggesting the absence of renal dysfunction at that time. Suspicion of nephropathy was raised due to the increased echogenicity of the renal cortex, proteinuria, and the sharp rise in Cr levels. The patient was subsequently transferred to another hospital for post-hospitalization support aimed at further evaluation of the rapidly progressing renal dysfunction and lymphadenopathy on hospital day 15.

Pathological examination 

**Figure 5:**
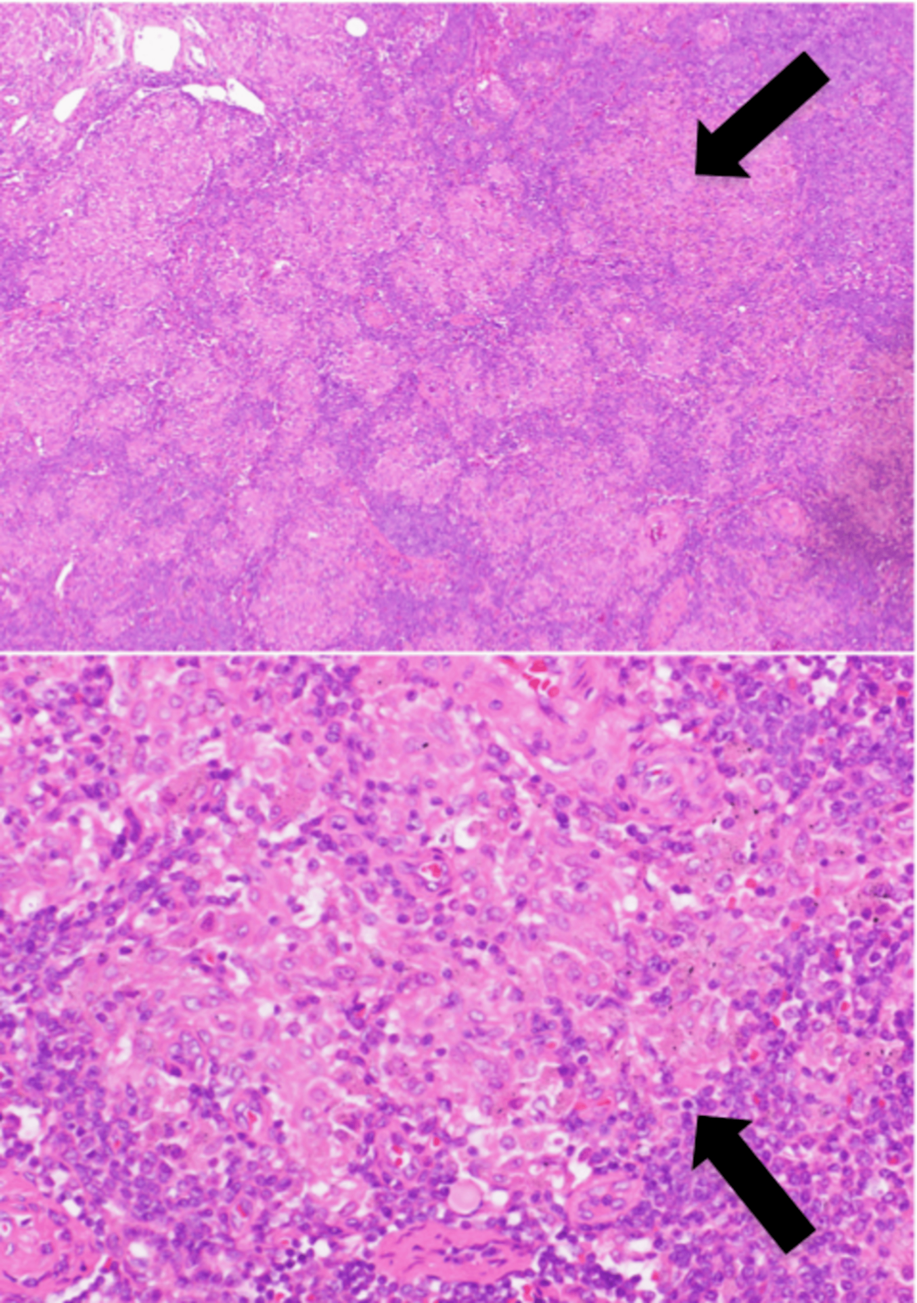
Pathological examination (×40) (×400) Here are the results of the renal biopsy. The upper panel shows a pathology image at 40x magnification, and the lower panel shows one at 400x magnification. Prominent sarcoid-like granuloma formation was observed (indicated by black arrows). No caseous necrosis was noted, and the appearance differed from that of typical tuberculous lymphadenitis.

Plain CT revealed enlarged para-aortic, left common iliac artery, external iliac artery, and inguinal lymph nodes. A lymph node biopsy was performed via the left groin, which indicated the presence of granuloma without caseous necrosis and multinucleated giant cells (Figure 5). Both acid-fast staining and fungal staining yielded negative results. No obvious signs of infection were observed. The soluble interleukin-2 receptor antibody and serum lysozyme levels were elevated, measuring 3290 U/mL and 30.6 μg/mL, respectively. A diagnosis of sarcoidosis was established. The angiotensin-converting enzyme (ACE) level was within normal limits, recorded at 18.3 U/L. Cardiac failure and renal failure were attributed to cardiac sarcoidosis and granulomatous interstitial nephritis. Long-term indwelling catheters were placed, and maintenance dialysis was initiated for renal dysfunction. After rehabilitation, the patient was discharged and returned home.

## Discussion

The risk of sarcoidosis is influenced by factors such as age, sex, and race, with incidence and prevalence varying significantly across different regions of the world. The prevalence of sarcoidosis is reported to be 60 per 100,000 people in the United States, 2.2 per 100,000 people in Taiwan, and 160 per 100,000 people in Sweden, illustrating considerable regional variation. The incidence ranges from 0.5 to 1.3 per 100,000 in East Asia, whereas it is notably higher in Scandinavian countries at 11.5 per 100,000 and in the United States at 7-11 per 100,000 [4-7]. A large-scale database study in the United States indicated that sarcoidosis is twice as common in women compared to men [4]. The condition typically manifests between the ages of 20 and 60 years, but more than half of patients are diagnosed at the age of at least 40 years [4,8]. Reports suggest that female patients are often diagnosed at an older age [9,10]. Our case involved an elderly female patient, whose age appeared to be slightly above the usual onset age for sarcoidosis. Furthermore, approximately 90-95% of patients present with lung symptoms; however, imaging in our case did not reveal any lung findings [6,11].

Research has identified several potential contributing factors, including occupational exposure, environmental influences, infections, and genetic predispositions, yet the exact cause remains unknown.

The incidence of concurrent renal disorders in patients with sarcoidosis, such as in this case, is reported to be low, at approximately 3% both in Japan and internationally [12]. The pathogenesis of renal disorders in sarcoidosis primarily includes the following: 1) conditions associated with abnormalities in calcium metabolism; 2) glomerular lesions related to systemic inflammatory diseases; and 3) the pathogenesis of granulomatous interstitial nephritis. Tissue diagnosis via renal biopsy is often required. In this patient, high serum calcium levels were not observed, and glomerular changes were minimal; however, she presented with tubulointerstitial nephritis accompanied by granuloma, indicating that the third factor is likely the primary cause of the aggravated renal function.

Regarding biomarkers, the study measured ACE, soluble IL-2 receptor antibody, and serum lysozyme; however, their clinical usefulness is limited [13]. Although ACE levels increase in 75% of patients with untreated sarcoidosis, the biomarker's sensitivity is low and its specificity is insufficient, resulting in a low diagnostic utility for serum ACE values [14-17]. Similarly, while soluble IL-2 receptor antibody concentrations are elevated in the serum of sarcoidosis patients, their diagnostic utility has not been definitively established [18]. Although the sensitivity of serum lysozyme in sarcoidosis is low, some studies have suggested that it may be suitable for prognostic evaluation [19,20]. Therefore, further research is warranted to identify more effective biomarkers for the future treatment of sarcoidosis.

In terms of treatment, corticosteroids are frequently employed for granulomatous interstitial nephritis, as seen in this case, and their effectiveness is well-documented [21]. Treatment is expected to improve renal function. However, corticosteroids were not administered to this patient because the symptoms unrelated to nephritis had already subsided by the time of diagnosis, and the patient’s advanced age of 89, along with concerns regarding potential adverse drug reactions, were taken into consideration.

Sarcoidosis should be considered and differentiated in cases where there is poor improvement in symptoms and laboratory data despite treatment for underlying conditions.

## Conclusions

We encountered a case of sarcoidosis diagnosed due to progressive renal dysfunction, lymphadenopathy, and cardiac involvement. While typical clinical presentations may indicate sarcoidosis, it is also crucial to consider the disease in the context of unexplained organ lesions. The administration of steroids must be evaluated carefully, considering the patient's long-term prognosis and relevant background factors.
